# Incidence and patterns of inborn errors of metabolism in the Eastern Province of Saudi Arabia, 1983-2008

**DOI:** 10.4103/0256-4947.65254

**Published:** 2010

**Authors:** Hissa Moammar, George Cheriyan, Revi Mathew, Nouriya Al-Sannaa

**Affiliations:** a Department of Pediatrics, King Faisal University, Dhahran, Saudi Arabia; b Pediatric Services Division, Saudi Aramco-Dhahran Health Center, Dhahran, Saudi Arabia; c Vanderbilt University, Tennessee, USA

## Abstract

**BACKGROUND AND OBJECTIVES:**

Individual inborn errors of metabolism (IEM) are rare disorders, but may not be that uncommon in our patient population. We report the incidence of IEM in a defined cohort of births at the Saudi Aramco medical facilities in the Eastern Province of Saudi Arabia over 25 years.

**METHODS:**

The records of all patients diagnosed with IEM from 1 January 1983 to 31 December 2008 were reviewed and categorized according to accumulated or deficient metabolites into small-molecule disorders (aminoacidemia, organic acidopathies [OA], urea cycle defects, fatty acid oxidation, and carbohydrate metabolic disorders) and other disorders, including glycogen and lysosomal storage disorders (LSDs), and organelle disorders.

**RESULTS:**

During the study period, 165 530 Saudi Arabian infants were born at Saudi Aramco and 248 were diagnosed with an IEM, corresponding to a cumulative incidence of 150 cases per 100 000 live births. Small-molecule disorders were diagnosed in 134/248 patients (54%). OA were the most common (48/248 patients; 19%), and methylmalonic aciduria was the most frequently observed OA (13/48 patients; 27%). LSDs were diagnosed in 74/248 patients (30%), and mucopolysaccharidosis was the most frequently observed LSD (28/74; 38%).

**CONCLUSION:**

We believe that our data underestimate the true incidence of IEM in the region. Regional and national newborn screening programs will provide a better estimation of the incidence of IEM. We recommend a centralized newborn screening program that employs tandem mass spectrometry.

Inborn errors of metabolism (IEM) can be classified according to the size of accumulated or deficient metabolites into small-molecule disorders such as aminoacidemia, organic acidopathies, urea cycle defect, galactosemia, fatty acid oxidation disorders, and other disorders such as glycogen and lysosomal storage and organelle diseases.^1^ Despite an increased understanding of their pathogenesis and prevalence, no uniform consensus on screening for these disorders has been established to date.^2^,^3^ Furthermore, the outcome of these disorders remains highly variable even with early diagnosis.

In countries where consanguinity rates are high, offspring can inherit 1/4 to 1/8 of their genes from a common ancestor, leading to an increased incidence of inherited disorders. The inbreeding coefficient factor ranges from 0.00004 to 0.0008 in Canada and from 0.001 to 0.005 in the United States. It is higher in Southern Europe, Latin America, and Japan.^4^,^5^ In the eastern province of Saudi Arabia, consanguinity rates are as high as 40% among first cousins and up to 60% in intermarriages between relatives, with an inbreeding coefficient factor of 0.024.^6^–^8^ The incidence of IEM in this region of Saudi Arabia has not been defined. In order to determine the specific IEM present in this population, as well as their incidence, we reviewed the medical records of all Saudi Arabian patients with a confirmed diagnosis of IEM born from 1 January 1983 to 31 December 2008, within the Saudi Arabian American Oil Company (Saudi Aramco) medical facilities.

## METHODS

Saudi Aramco provides comprehensive free healthcare for its 55 000 employees and their 370 000 dependents at several health care facilities and a major 400-bed tertiary care hospital in Dhahran. This review covers a 25-year period from 1 January 1983 to 31 December 2008.

### Birth Cohorts

The data on Saudi Arabian infants born during the 25-year period were taken from the Mortality and Morbidity Reports 1983-2008, Epidemiology Services Unit, Preventive Medicine Services Division, Saudi Aramco Medical Services Organization (SAMSO)·^9^ During the study period, 165 530 live births were recorded, and 248 patients were diagnosed with various forms of metabolic diseases. These patients were diagnosed and followed-up in the main medical center at Dhahran.

Apart from classic phenylketonuria, for which newborn screening was started in Saudi Aramco in 1980,^10^ the remaining patients were evaluated on the basis of their clinical manifestations and/or family history of either unexplained neonatal death or previously affected members. Almost all the parents of the affected patients were consanguineous. The number of affected children varied from one to nine per family, with a mean of two, which facilitated the selective screening of all siblings born to previously identified families with metabolic disease. Autopsies were not available for the deceased patients.

### Confirmation of Diagnosis

The initial biochemical investigations included analysis of quantitative plasma and cerebrospinal fluid amino acids, plasma acylcarnitine profiles, urine organic acids, oligosaccharidoses, and glycosaminoglycans. Plasma very long-chain fatty acids, phytanic, and pristanic acids were also assessed for the diagnosis of peroxisomal disorders. For urea cycle disorders, diagnosis was based on the presence of hyperammonemia and the typical plasma amino acids profiles. The diagnosis was confirmed by enzyme activity estimation on either cultured skin fibroblasts or in the blood of all patients with fatty acid oxidation disorders. Biotinidase deficiency and 3-methylcrotonyl aciduria were diagnosed by blood enzyme assay and skin biopsy, respectively. Tetrahydrobiopterin biosynthesis defects secondary to a pyruvoyl tetrahydrobiopterin synthase deficiency was diagnosed by measuring the level of neurotransmitters in the cerebrospinal fluid, followed by confirmation of the diagnosis by a mutation study. Three (3/6) patients with propionic aciduria, 11 patients (11/14) with methylmalonic aciduria, 1 patient (1/5) with tyrosinemia, and 1 patient (1/12) with maple syrup urine disease (MSUD) had their diagnoses confirmed by enzyme assay. The diagnosis of all glycogen and lysosomal storage disorders, apart from neuronal ceroid lipofuscinosis (NCL), was confirmed by enzyme activity estimation on cultured skin fibroblasts, liver biopsy, or leukocytes. Niemann-Pick disease was diagnosed by conventional filipin staining and the absence of cholesterol esterification in cultured skin fibroblasts in all patients.

Genotyping identified all families with biopterin biosynthesis defect; tyrosinemia; short chain acyl-coenzyme A dehydrogenase (SCAD), carnitine palmitoyl transferase I (CPTI), carnitine palmitoyl transferase II (CPTII), 3-phosphoglycerate dehydrogenase (3PGD), and carbonic anhydrase II (CA II) deficiencies; carnitine uptake defect (CUD); galactosemia; mucopolysaccharidosis (MPS) I; Fabry disease; gangliosidosis (GMI); Niemann-Pick disease type C (NPC); and glycogen storage disease (GSD) type Ia and GSD type II.

Mithochondrial DNA studies included multiplex polymerase chain reaction amplification of the relevant segments of the mitochondrial DNA genome followed by dot blot hybridization with allele-specific oligonucleotide probes to evaluate deletions or rearrangements. The entire mitochondrial DNA was not sequenced in these patients and specific targeted mutations of mitochondrial DNA need further study.

The biochemical studies, including quantitative serum amino acid analysis, acylcarnitine profiles, carnitine and urine analyses for organic acids, oligosaccharides and glycosaminoglycans analyses, were carried out at the Bio-Sciences Laboratory, San Diego, California from 1983 to 1991 and the Mayo Clinic Laboratory, Rochester, Minnesota from 1991 to 2008. Enzyme activities were tested for lysosomal storage diseases at either Willink Biochemical Genetics Laboratories, Manchester, UK, or the Mayo Clinic Laboratory, Minneapolis, USA.

All cases were reviewed for the following variables: gender, previous family history of IEM, diagnosis, outcome, and follow-up. Incidence was calculated by dividing the number of diagnosed cases by the number of total births during the study period. Disease-specific numbers per 100 000 were calculated by dividing the number of cases by the total population and multiplying by 100 000.^11^

## RESULTS

Over the 25-year period of this retrospective study, 248 patients were diagnosed with 55 metabolic diseases. Tables 1 and 2 illustrate the distribution of patients diagnosed with small-molecule and other forms of IEM, respectively. The tables indicate the estimated incidence per 100 000 live births for each group of disorders.

**Table 1:** Incidence data for disease categories. Small-molecule disorders of IEM in SAMSO (1983-2008). Total population of live births (165 130).

Disease category	Numbers of cases diagnosed	No. of families affected	Incidence rate/100 000 live births
Aminoacidopathies	38	18	23
Classic homocystinuria	4	3	2
NK hyperglycinemia	1	1	1
Biopterin biosynthesis defect	4	3	2
Classic PKU	12	3	7
Hepatorenal tyrosinemia	5	1	3
MSUD	12	7	7
Organic acidopathies	48	29	29
Propionic aciduria	6	3	4
Methyl malonic acidemia			
Cobalamin B deficiency	9	3	5
Mutase deficiency	2	2	1
Unknown	3	3	2
Isovaleric aciduria	6	3	4
3-Methylcrotonyl aciduria	3	2	2
2-Hydroxyglutaric aciduria	8	4	5
Glutaric aciduria type I	3	2	2
Multiple carboxylase deficiency	2	3	1
Biotinidase deficiency	3	3	2
Canavan disease	3	1	2
Urea cycle disorders	12	4	7
Citrullinemia	6	2	4
Argininosuccinase deficiency	6	2	4
Fatty acid oxidation disorders	18	9	11
SCAD deficiency	4	2	2
MCAD deficiency	2	1	1
LCHAD deficiency	1	1	1
CPT I deficiency	2	1	1
CPT II deficiency	4	1	2
CACT deficiency	3	1	2
Carnitine uptake defect	2	2	1
Carbohydrate disorders	16	10	10
Galactosemia	16	10	10
Serine deficiency	2	1	1
3 PGD deficiency	2	1	1

NK: nonketotic; PKU: phenylketonuria; MSUD: maple syrup urine disease; SCAD: small-chain acyl-coenzyme A dehydrogenase; MCAD: medium-chain acyl-coenzyme A dehydrogenase; LCHAD: long-chain 3-hydroxyacyl co-enzyme A dehydrogenase; CPT: carnitine palmitoyl transferase; CACT: carnitine-acylcarnitine translocase; PGD: phosphoglycerate dehydrogenase.

**Table 2:** Incidence data for disease categories: glycogen storage, lysosome storage, mitochondrial, and other IEM disorders in SAMSO faciltities (total population of live births: 165 530.

Disease category	Numbers of cases diagnosed	No. of families affected	Incidence rate /100 000 live births
Glycogen storage diseases	17	10	10
GSD type I A	4	2	2
GSD type I B	1	1	1
GSD type II	3	1	2
GSD type III	8	5	5
Fanconi-Beckel syndrome	1	1	1
Lysosomal storage diseases	74	42	44
Mucopolysaccharidosis			
MPS I	6	2	4
MPS III	3	2	2
MPS IV	6	4	4
MPS VI	13	7	8
Niemann-Pick A	8	6	5
Niemann-Pick C	2	2	1
Fabry disease	9	1	5
Sandhoff disease	9	7	5
NCL (Juvenile)	9	5	5
Gangliosidosis (GMI)	4	2	2
Galactosialidosis	1	1	1
Manosidosis	2	1	1
Wolman disease	1	1	1
Cystinosis	1	1	1
Organelle disorders	18	6	11
Mitochondrial			
PDH Deficiency	3	2	2
Suspected mitochondrial disorders			
Leigh’s disease (undetermined enzyme)	7	6	4
Congenital lactic acidemia	4	4	2
Peroxisomal			
Peroxisomal biosynthesis defect	1	1	1
DHAPAT Deficiency	1	1	1
Dihyrdoacetone phophate			
acyltransferase deficiency (rhizomelic chondrodysplasia punctata type 2	2	2	1
Other disorders	5	3	3
CA II deficiency	3	2	2
DPD deficiency	2	1	1
All inborn errors of metabolism	248	142	149

NCL: neuronal ceroid lipofuscinosis; PDH: pyruvate dehydrogenase deficiency; CAII: carbonic anhydrase II; DPD: dihydropyrimidine dehydrogenase deficiency.

Lysosomal storage disorders (LSDs) were the most frequently diagnosed family of IEM and were observed in 74/248 patients (30%). Mucopolysaccharidosis (MPS) constituted the largest subgroup of LSDs (28/74 patients; 38%), and MPS IV was the most prevalent type of MPS, observed in 13/28 patients. Organic acid disorders were the second largest IEM group, affecting 48/248 patients (20%). Methyl malonic aciduria (MMA) constituted the largest subgroup of patients with organic acidurias (14/48 patients), and the majority of MMA cases were secondary to cobalamin B deficiency (9/14 patients).

Amino acid disorders were diagnosed in 38/248 patients (16%). Fatty acid oxidation disorders were diagnosed in 18/248 patients (7%) and were represented by different enzymes deficiencies. Genetic analysis for medium-chain acyl-coenzyme A dehydrogenase (MCAD) patients failed to identify the common mutation 985 A>G. Only 12/248 patients (5%) were diagnosed with urea cycle disorders.

For patients with suspected mitochondrial disorders, the diagnosis of pyruvate dehydrogenase deficiency (PDH) with typical clinical manifestations of Leigh disease was confirmed in three patients by enzyme assay of cultured skin fibroblast. The mitochondrial respiratory chain enzyme study performed on these three patients was not conclusive, and none underwent a muscle biopsy or nuclear DNA mutation analysis. Mitochondrial DNA was normal in all of the patients tested. Two patients with a mitochondrial disorder were first double cousins from consanguineous families with more than one affected member. The third patient, who had a Saudi Arabian father and a Filipina mother, was confirmed to have an X-linked PDH-E-1a subunit deficiency.

Ninety patients (90/248; 36%) were siblings of index cases. During the latter half of the study period, data on all families diagnosed with metabolic disease were available on a computer database. Table 3 provides a comparison of the major IEM defects observed in retrospective international population-based studies. The comparison of those reports with our study reveals a high incidence of IEM in our population, especially for LSDs, organic acidopathies, and aminoacidopathies.

**Table 3:** Comparison of frequencies of IMD and categories of disorders found by retrospective population-based studies.

Program	SAMSO-DHC	British Columbia 1	Italy 20	West Midlands 22	Oman 21
Study years	1983-2008	1969-1996	1985-1997	1999-2003	6/1998-12/2006
Population	165 530	1 142 912	7 173 959	310 510	51 000
No. of cases	248	173	1935	396	82
General	1:667	1:2500	1:3	703 (1:2 758)^b^	1:784	1:1555
Aminoacidopathies	1:4356	1:6606	1:36 389	1:5354	1:6375
Glycogen storage disease	1:9737a	1:69 054^a^	1:19 532	1:14 786	1:7285
Galactosemia	1:10 345	1:36 200	1:50 316^c^	1:16 343	1:25 500
Fatty acid oxidation disorder	1:41 382	NA	1:91 599	1:12 938	1:7285
Lyosomal storage diseases	1:2236	1:13 112	1:8 275	1:5 175	1:2318
Urea cycle disordersa	1:13 794	1:53 717	1:41 506	1:22 179	1:6375
Organic acidopathies	1:3448	1:27 082	1:21 422	1:7962	1:5666

a Total including Pompe disease;

b In last 5 years of study;

c Regional Neonatal Screening.

## DISCUSSION

The spectrum and incidence of metabolic disease in this retrospective study covers a well-defined population in the eastern province of Saudi Arabia. Currently, we selectively screen newborns in families with a known IEM disorder and initiate appropriate medical intervention. We provide genotype testing to identify the disease-causing mutations in affected families. Furthermore, we offer asymptomatic carrier testing and pre-natal genetic diagnosis for those families. Thus, the high incidence rate of disease we observed strongly supports the expansion of current newborn screening to include other IEM.

Over 25 years, 248 patients with 55 different metabolic diseases were identified. Several case reports from Riyadh, Saudi Arabia, have indicated that certain diseases are confined to specific families and tribal groups. 12–22 Additionally, the frequency of metabolic disorders (1:667) in this retrospective series is not strictly comparable to studies based on neonatal screening results because those programs do not test for all the disorders analyzed in the present study. Nevertheless, it revealed an incidence of IEM higher than that reported in the literature.^1^,^10^–^24^

In our series, LSDs comprised the most frequently occurring group of disorders, with an estimated combined incidence of 1:2236. LSDs were observed in 45/100 000 live births, or 31.3% of all IEM diagnoses. The frequency of LSD that we observed is much higher than that reported in Australia,^25^–^27^ British Columbia,^1^ Germany,^19^ Italy,^20^ West Midlands UK,^22^ the Netherlands,^28^ and the USA.^23^ Coelho et al^29^ also reported a high incidence of LSD (59.8%) in Brazilian patients referred for confirmation or exclusion of LSD. Specifically the incidence of MPS in our series, i.e. 16.9/100 000 live births (7%), is also much higher than that reported from other countries.^1^,^9^–^21^,^30^,^31^ MPS type VI was the most common type in our series, with an incidence of 7.8/100 000 births. Organic acidopathies were the most prevalent group of small-molecule disorders, with a higher incidence (1:3448) than that reported in Australia,^18^ British Columbia,^1^ Germany,^19^ Italy,^20^ Kuwait,^24^ Oman,^21^ West Midlands UK,^22^ and the USA,^23^ but lower than that reported from Riyadh, Saudi Arabia.^17^ In contrast, among the fatty acid oxidation (FAO) disorders, MCAD deficiency, one of the most commonly reported disorders in previous studies^3^,^32^–^39^ had a much lower incidence in this report (1:82 765). Similarly, Han et al reported a lower incidence of FAO disorders in a Chinese population in a 4-year retrospective review of clinically selected cases.^40^

Galactosemia was identified in six Saudi couples between 1983 and 1994, as determined from SAMSO records from the eastern province of Saudi Arabia, with a corresponding incidence of 1:8500 live births.^41^ Although 3 of those families were lost during the follow-up, we still observed 16 patients with galactosemia from 10 families. The corresponding incidence of 1:10 345 live births remains higher than that reported in other countries.^1^,^42^–^44^

Mitochondrial diseases represented a unique situation in our cohort in that none of our patients were identified to have mitochondrial DNA mutations, which is consistent with the autosomal recessive mode of inheritance and suggestive of nuclear rather than mitochondrial DNA mutation.^45^,^46^ Three patients were confirmed to have PDH deficiency on cultured skin fibroblasts. The remaining seven patients had typical clinical manifestations of Leigh disease with lactic acidosis and central nervous system changes. Mitochondrial respiratory studies were performed on cultured skin fibroblasts only and were unable to identify the underlying biochemical defects.

Our data strongly justify the expansion of a newborn screening program to include treatable conditions such as LSD, organic acidopathies, aminoacidemia and galactosemia, with standardized protocols for evaluation and treatment of identified newborns. Along with genetic counseling, this will substantially enhance the quality of care provided to this group of patients. Newborn screening is one of the most successful public health programs to facilitate early detection and management of IEM as well as to prevent mortality and morbidity in affected children.^47^–^50^ We recommend a centralized, country-wide newborn screening program utilizing tandem mass spectrometry.^51^,^52^ A coordinated follow-up and treatment plan for the selective treatable disorders could be set up regionally, and genetic counseling made available to families where no intervention is currently possible.
